# Custom-Made Antibiotic Cement Nails in Orthopaedic Trauma: Review of Outcomes, New Approaches, and Perspectives

**DOI:** 10.1155/2015/387186

**Published:** 2015-10-05

**Authors:** Marcin K. Wasko, Rafal Kaminski

**Affiliations:** ^1^Department of Orthopaedics and Rheumoortopaedics, Prof. A. Gruca Teaching Hospital, Konarskiego 13, 05-400 Otwock, Poland; ^2^Department of Trauma Surgery and Orthopaedics, Prof. A. Gruca Teaching Hospital, Konarskiego 13, 05-400 Otwock, Poland

## Abstract

Since the first description in 2002 by Paley and Herzenberg, antibiotic bone cement nails (ACNs) have become an effective tool in the orthopaedic trauma surgeons' hands. They simultaneously elute high amounts of antibiotics into medullary canal dead space and provide limited stability to the debrided long bone. In this paper, we perform a systematic review of current evidence on ACNs in orthopaedic trauma and provide an up-to-date review of the indications, operative technique, failure mechanisms, complications, outcomes, and outlooks for the ACNs use in long bone infection.

## 1. Introduction

Musculoskeletal infections remain a challenge for orthopaedic surgeons and infectious disease specialists. Bone provides a unique milieu for bacteria, with low vascularity and turnover rate. Most of the orthopaedic trauma infections are caused by biofilm-forming bacteria [1]. Biofilm consists of hydrated matrix of polysaccharide and protein. Once formed, it protects the microorganism from antimicrobials, opsonization, and phagocytosis, thus contributing to the chronicity of infections [2]. In order to cure biofilm-related infection, four principles formulated by Cierny and Mader must be observed: (a) complete surgical debridement with dead space management, (b) fracture/nonunion stabilization, (c) soft tissue coverage, and (d) adequate antibiotic levels [3]. In healthy bone, local antibiotics' concentrations might be less than 20% of serum levels, as is for most beta-lactams [4]. Their efficacy is further diminished by biofilms, which decrease molecule penetration [5]. With intramedullary infections, the optimal way of debridement is to ream the medullary canal. After reaming, it takes approximately 4 weeks for bone to revascularise [6]. Therefore, even with prolonged antibiotic therapy, local bone tissue remains without bactericidal concentrations, thus not interfering with bacterial growth. Acrylic bone cement is the gold standard for dead space management and the standard carrier for local antibiotic delivery in the management of orthopaedics infections [2, 7]. It delivers high concentrations of the drug locally, even to avascular areas that are inaccessible to systemic antibiotics. Those concentrations are high enough to be effective even against organisms that are resistant to drug concentrations achieved by intravenous supply. At the same time, very low serum antibiotic concentrations are observed and hence the risk for toxicity is considerably diminished [8, 9].

**Figure 1:**
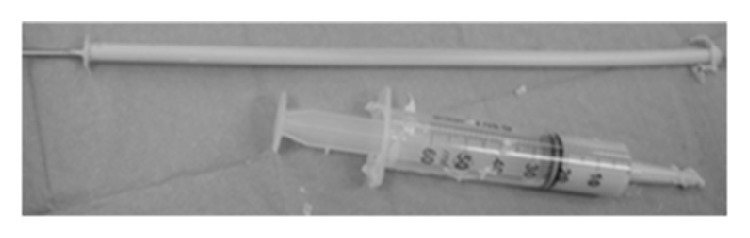
The photo of the prepared intraoperatively* K*-wire-armed, antibiotic loaded cement nail with a syringe (for scale comparison).

Antibiotic loaded bone cement can be customized intraoperatively to different shapes and forms. In intramedullary infections, antibiotic bone cement nails or antibiotic cement nails (ACNs) are preferable.  Figure 1 presents an example of an ACN. They offer local delivery of antibiotics, while filling the dead space and offering stability to the fracture/nonunion site, if present.

The primary objective of this paper is to perform a systematic review of current evidence on ACNs in orthopaedic trauma. The secondary objective was to provide an up-to-date review of the indications, operative technique, failure mechanisms, complications, outcomes, and outlooks for the ACNs use in musculoskeletal infection.

## 2. Materials and Methods

### 2.1. Search Strategy

To identify relevant papers, we searched Medline database via PubMed interface with no restriction on language or publication date. The search string included word “intramedullary” and one of the following words, “osteomyelitis,” “infection,” “nail,” “nailing,” “debridement,” and “reaming” and was performed on January 1, 2015. We searched all fields in the Medline database, with no restriction applied to full-text availability. Additionally, we manually reviewed the reference lists of the articles retrieved by database search for potentially missing papers.

The clinical studies selected were original articles on antibiotic cement nails. We excluded all the studies on bone cement use in arthroplasty and spine surgery as well as* in vitro* and* in vivo* studies, although we did not use the “restrict to human studies” filter in PubMed.

Details such as the number of patients, anatomical site, the age of the patients, the preparation and composition of nail, length of follow-up, and final outcome were collected.

## 3. Results and Discussion

### 3.1. Search Results

**Figure 2:**
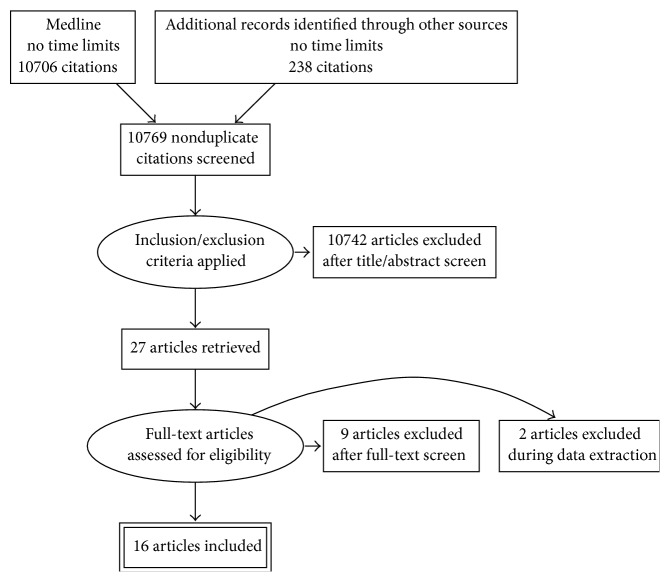
Article search strategy flow diagram for this paper.

Literature review of antibiotic cement nails cases is given in Table 1.  Figure 2 shows search strategy flow diagram. Since detailed discussion of clinical outcomes is beyond the scope of this review, for detailed discussion of clinical outcomes and perspectives on intramedullary infection treatment, including ACNs, we suggest the reader consults an excellent clinical review by Makridis et al. [10].

### 3.2. Indications for ACNs

The indication for the use of ACN is medullary infection, whatever the cause and presentation. Intramedullary infection is a well-recognized complication of intramedullary nailing for trauma [11]. It spreads along the length of the nail and involves the entire length of bone [12]. Multiple points or also the entire medullary canal is involved in pin tract infections after external fixation [13]. Reported rates of infectious complications after planned conversion from external fixation to intramedullary nailing for the femoral fractures range from 1.7 to 20% [14–18]. Similar problems can occur with lengthening over nails with external fixation and transport over nail [19, 20]. Therefore, the indications for ACN span from long bone fractures with concomitant soft tissue damage, to infected nonunion sequelae of external fixation and haematogenous osteomyelitis.

All these diagnoses share a common trait; there is usually no sequestrum and dead bone is limited to within the medullary canal [21]. However, according to the Cierny principles, after the removal of the intramedullary nail it acts as an avascular noncollapsible dead space that needs to be managed [22]. It could not be managed appropriately with poly(methyl methacrylate) (PMMA) beads strung on elastic wire and introduced into medullary canal, since they do not conform to the dead space shape and their removal becomes increasingly difficult starting already 2 weeks after insertion due to fibrous overgrowth [23]. Rather than using multiple beads on a single wire, Klemm and in another paper Seligson and Klemm used PMMA stick, formed of a PMMA mass attached to a single wire [24, 25]. This construct could be passed around external fixator's pins and its removal was facilitated. Unfortunately, it did not provide any mechanical stability to the fracture/nonunion site. In that scenario, stability is very important to treat the infection and to obtain drainage cessation or lessening [26, 27].

### 3.3. Contraindications for ACNs

There is one paper stating that, in bone deficits exceeding 6 cm, other alternatives for restoring stability and infection control should be used [34]. There is also an obvious contraindication for the reaming and, thus, the use of nail in people under 16 years of age [28].

### 3.4. The Role of Local Antibiotics Delivery versus the Role of Debridement

In a 2013 Cochrane review, the authors could not show any significant difference in osteomyelitis recurrence rates after parenteral or oral antibiotic administration [39]. Although it might be due to the fact that only limited and low quality evidence on the subject is available, this might also suggest the importance of surgical debridement as a cure, which is an observation commonly accepted among the orthopaedic surgeons [40]. A prospective trial by Simpson et al. documented the effect of surgical debridement on cure rates. They achieved 100% success with wide excision (clearance margin of 5 mm or more), 28% failure rate with marginal resection, and a total failure with local debulking and intralesional biopsy [41]. The importance of this issue is highlighted by articles describing novel ways to thoroughly debride the medullary canal, for example, RIA or Pressure Sentinel [42]. Therefore, it must be borne in mind that all the clinical results of ACNs show at the same time the results of debridement and other therapeutic actions [43].

### 3.5. Nail Fabrication

Multiple techniques for the fabrication of the nails have been described, from manual rolling of the cement [34], through the use of chest tube as a temporary mould, which is peeled off once the cement hardens [12, 21, 23, 29, 35, 37, 38, 44], to using a reusable mould [45, 46].

Usually, a chest tube or another kind of drainage tube with an inner diameter similar to the outer diameter of the removed nail or the diameter of the last reamer used is selected and closed at one end, for example, with Kocher forceps. Some kind of guide wire (*K*-wire, Ender nail, etc.) is selected and cut a little bit longer than the tube and its end is bent to facilitate later extraction from the medullary canal. It can also be bent to adapt to the dead space, for example, with the Herzog angle in case of replacing a tibia nail. Later, antibiotic powder is mixed with poly(methyl methacrylate) powder. The next step is to add liquid monomer, usually more than would be used for the single batch of cement, to compensate for large volume of antibiotics. Next, the cement is poured into the cement gun and injected into the tube. The precut wire is inserted into the middle of the cement in the tube. At some point, either when the cement begins to harden and heats up or after the exothermic reaction, the tube is cut with surgical knife and peeled from the cement. Then the surgeon waits for the nail to cool and for the monomer to evaporate, which usually takes around 15 minutes. The nail can then be inserted into the medullary canal, with the bent/looped end extruding for easier retrieval.

### 3.6. Cement Type

When compared with other bone cements, Palacos cement showed highest elution rates, which was explained by its inherent porosity [48].

### 3.7. Mixing Techniques

It has been advocated that vacuum mixing the cement results in stable antibiotic elution [21], although this effect varies for different cement types [49]. On the other hand, there are papers arguing that vacuum mixing decreases cement's porosity and thus reduces the total elution of the antibiotic [50]. In-depth analysis was provided by Neut et al., who showed that vacuum-mixed gentamicin-loaded Palacos R released more gentamicin after one week* in vitro *than hand-mixed Palacos R did despite a reduction in the porosity, which theoretically should reduce elution [51]. The authors explained this discrepancy by the increase in the number and distribution of micropores smaller than one millimetre in the vacuum-mixing group, which occurred during cement polymerization by evaporation of the volatile monomer. On the other hand, the same article mentions that hand mixing with a spatula resulted in increased antibiotic release than hand mixing in a dedicated system (Cemvac) [51]. It has also been brought to attention that mixing systems in general are very heterogeneous in regard to resulting cement porosity, which probably influences the elution rates a great deal [52, 53].

### 3.8. Antibiotics

#### 3.8.1. Antibiotics: General Considerations

To effectively eliminate bacteria in a biofilm, local antibiotic concentrations achieved must be 10 to 100 times the usual bactericidal concentration [5]. This usually cannot be achieved by safe doses of parenteral antibiotics, rendering this form of biofilm treatment ineffective [2]. Bone cement can deliver high concentrations of antibiotics, even to poorly vascularised and hypoxic environment, as it is independent of vascular supply [21, 54–56]. For the most popular antibiotics, bactericidal concentrations were found for up to six weeks after implanting PMMA beads [57]. Most likely, this observation would be applicable to the use of nails, since hip spacers show similar pharmacokinetics [58].

The antibiotics used for the fabrication of ACNs should be available in powder form and have a wide antibacterial spectrum with bactericidal activity at low concentrations [59]. Other desirable characteristics include high elution rate from PMMA over long periods of time and thermal stability with low influence for the mechanical properties of the cement [8]. Since they are eluted locally, they should not cause allergy or bind to serum protein easily, to maintain high concentration [48, 54]. They should also have as minimal as possible inhibitory influence on new bone formation [60].

Liquid antibiotics elute larger amounts from bone cement than antibiotics in powder form [8]. However, they are not used in clinical practice since they negatively influence the mechanical properties of PMMA [61, 62]. Antibiotics in powder form are reported to have a rather negligible effect on the mechanical stability of bone cement as long as the antibiotic to cement ratio does not exceed 10%, which is a lower proportion than usually applied in the ACNs formulation [63].

#### 3.8.2. Antibiotic Choice: Microorganism Sensibility

Nowadays, effective serum levels describe sensitivity of bacteria to the antibiotics. Most bacteria defined as resistant by these criteria might be sensitive when exposed to local antibiotics [7, 55, 64]. To guide the antimicrobial treatment properly, pathogens should be reconsidered to be either sensitive or resistant to the antibiotic levels achievable locally [7, 31, 55]. This is the case, for example, in classical Buchholz's work, where the antibiotic placed in the bone cement did not necessarily correlate with the culture-based sensitivity of the organisms [65]. On the other hand, there have been reports of changing patterns in microorganism resistance, partly as a result of widespread local and systemic prophylaxis [66]. Increasing numbers of gentamicin-resistant species are reported to cause deep infections, including medullary infections [67, 68]. One must remember that, even with antibiotics' concentrations as high as after adding up to 20% weight of the PMMA, colonization of the spacer/nail can still occur [69].

#### 3.8.3. Antibiotics: Dosage

Acrylic bone cement was primarily developed as a fixation device for arthroplasty. Although the addition of more than two grams of any antibiotic per 40 g of cement reduces the cement's strength, this is not relevant to the infection treatment, as those devices are only temporary and only partially loaded mechanically [56]. To maintain elution rates and concentrations sufficient to treat an established musculoskeletal infection, at least 3.4 g of antibiotic should be used for 40 g batch of PMMA [70, 71].

#### 3.8.4. Antibiotics: Elution

There are no papers on antibiotic elution from ACNs in English. The only report we were able to find was a Japanese article with no English translation [72]. However, there is a multitude of antibiotic elution studies on cement beads and spacers. Although the absolute values for drug release from ACNs might be different from beads and spacers, the general proportions between elution rates and processes governing the elution remain the same [73].

The first study on elution was performed by Marks et al., who showed that oxacillin, cefazolin, and gentamicin elute in a microbiologically active form from Palacos and Simplex bone cements [62]. Afterwards, it was established that elution of antibiotics from acrylic cement follows a biphasic pattern, with high elution rates early, followed by slower but sustained elution rates as time progresses [8]. Only a small portion of the antibiotic incorporated in bone cement is released; the amount of antibiotic released from cement shows an exponential decline after day 1 of implantation [49, 51, 74]. The hydrophobicity of ALBC permits roughly 10% of the added antibiotic to elute over a 6–8-week period [8].

The mechanism by which these drugs are released is not fully understood. First papers to tackle that issue suggested that the elution of antibiotics from bone cement was mainly by diffusion [75, 76]. The diffusion theory relies on the presence of pores and interconnected channels in bone cement, through which the circulating medium penetrates and dissolves the incorporated antibiotics which then slowly diffuse outwards [77]. However, Masri et al. argued that the data provided by the diffusion theory protagonists did not support this conclusion [78].

Nowadays, most of the research suggests that antibiotic release from PMMA is a passive phenomenon in which diffusion occurs out of pores, cracks, and voids in the cement [8, 79]. In support of this theory, studies show that elution is improved with increasing surface area and porosity of the cement. [62, 78–80]. van de Belt et al. studied the release of gentamicin as a function of time from six different gentamicin-loaded bone cements. They related elution to surface roughness, porosity, and wettability of the PMMA. The release kinetics of gentamicin in their study was controlled by surface roughness and porosity. They suggested that the initial release antibiotic from bone cement was mainly a surface phenomenon, while sustained release over several days was a bulk diffusion phenomenon [81]. This theory best accounts for the biphasic release characteristics of antibiotic bone cement [8].

The elution of antibiotic from PMMA would be therefore dependent on multiple variables: which cement is used [48, 54], which antibiotic is chosen [71], the amount of total antibiotic added [71], and how it is mixed [82]. Highly porous cement has been shown to elute more antibiotic and for a longer period of time relative to cement with less porosity [71].

Commercially manufactured antibiotic loaded drug delivery systems have more predictable elution patterns, compared to devices manufactured with a manual addition of an antibiotic (due to the more homogenous distribution of the incorporated antibiotic(s) in the cement powder). However, this is often the only way to make the bone cement appropriate to the sensitivity profile of the causative pathogen. Adding another antibiotic powder not only increases the activity spectrum but also increases the antibiotic elution rates. Most studies evaluating combinations of antibiotics have demonstrated a synergistic effect, in that adding a second antibiotic seems to increase the elution of both antibiotics [8, 54, 71, 83]. This is true for tobramycin and vancomycin [71, 84], teicoplanin and gentamicin [83], and linezolid and gentamicin [85]. However, two studies have reported that combining antibiotics results in decreased elution [86, 87]. No change in elution rate was reported when vancomycin and amikacin had been used together; elution rates were the same as when used individually [73]. On the other hand, with the use of another PMMA cement, different antibiotics ratio, and different testing conditions, one can find that release of one of the drugs is inhibited by the addition of another antibiotic [87]. Generally, when two antibiotics are mixed into the same batch of bone cement, a phenomenon called passive opportunisms occurs; one of the substances acts as a soluble additive increasing porosity of the cement and increasing the total elution [71]. Thus, total antimicrobial effect of eluted substances is increased. This phenomenon seems to depend on the volume ratio between two antibiotics added to PMMA [8].

Díez-Peña et al. tried to predict the gentamicin elution from low- and high-loaded bone cement (containing up to 10 wt% of gentamicin sulfate and more than 20 wt% of gentamicin sulfate in relation to the weight of entire block of bone cement) by means of mathematical equations in an experimental model. They found out that each differently loaded PMMA has a different equation describing the release process. Moreover, for low-loaded cement, the release was mainly controlled by the imperfections of the matrix, whereas in the high-loaded PMMA an abrupt increase in the amount of drug released was evident allowing the almost complete release of the drug incorporated [88].

Lastly, the method used to mix the antibiotic seems to play an important role. Hand mixing with a spatula has been shown to increase the total antibiotic release when compared with mixing with a specialized cement mixer [51]. It is hypothesized that hand mixing introduces significant porosity to the cement, which in turn should increase antibiotic elution [8]. Other authors argue that hand mixing, in contrast to device mixing, does not crush the antibiotic crystals and thus may improve elution characteristic [50].

#### 3.8.5. Antibiotics: Current Practice

The most used antibiotic for the fabrication of ACNs is gentamicin, followed by vancomycin [30, 44]. Concomitant use of these two agents can widen the spectrum of activity but also enhance the elution rates for both substances simultaneously [89].

#### 3.8.6. Antibiotics: Complications

Only low serum concentrations and minor systemic toxicity are achieved while using ACNs and other local cement delivery devices [54, 90]. Research has consistently shown that antibiotics added to cement devices do not reach significant concentrations in the bloodstream, and there is no systemic toxicity in otherwise healthy individuals without hepatic or renal disease [55]. We found four case reports that have been published on acute renal failure after the use of high-dose ALBC for the treatment of deep periprosthetic sepsis, three total knees and two total hip patients [91–94]. Concurrent administration of IV antibiotics and significant comorbidities (e.g., preexisting renal disease) complicate all of these patient histories. On the other hand, a study from Mayo clinic by Springer et al. which retrospectively reviewed 36 knees in 34 patients treated with 4 g of vancomycin and 4.8 g of gentamicin per 40 g batch of PMMA reported only a single case of transient elevation in serum creatinine with no permanent systemic complications [95]. Noteworthily, the mean total dose of antibiotic per patient in that study was 10.5 g of vancomycin and 12.5 g of gentamicin, which is much higher than with any of the ACNs reported. Up to date, we were not able to find any report on renal or auditory complication in ACN patients. This might be due to the fact that the majority of those nails are put in trauma or posttraumatic patients, who tend to be younger and previously healthy compared to, for example, total joint replacement patient population. Multiple papers show that patients treated with local antibiotics are at no more risk and are probably at less risk of experiencing ototoxicity and nephrotoxicity than ones subjected to long-term parenteral antibiotics [96–98]. There are more adverse reactions due to the use of systemic antibiotics than to the use of local antibiotic-eluting spacers and nails [99].

Another important complication of ACNs is resistance induction. There are two articles describing ability of bacteria to adhere to and colonize gentamicin [69] and mixed gentamicin-vancomycin-loaded cement [100] after prolonged periods of implantation and even ability to develop gentamicin resistance despite preoperative susceptibility.

### 3.9. *In Vitro* and* In Vivo* Studies on ACNs

As far as the authors know, there is no report on the use of ACNs in an animal model. Most probably this is due to the technical difficulties and availability of similar models, that is, antibiotic-coated implants. Local application of a biodegradable coating with 10% gentamicin was shown to be effective in reducing implant-related infection in a rat model [101]. Similar data was found for fibrin sealant plus tobramycin, a combination which was as effective as poly(methyl methacrylate) beads plus tobramycin against methicillin-sensitive* Staphylococcus aureus *osteomyelitis in a rabbit model [102].

Elson et al. performed one of the most important early laboratory studies. They showed that when antibiotic loaded acrylic bone cement is placed next to cortical bone, dense cortical bone is penetrated by the eluted antibiotic and its concentration in the bone is much higher than what can be achieved safely by systemic administration [103].

### 3.10. ACNs Benefits and Risks

The benefits and risks of ACNs are summarised in Table 2. Since the benefits were thoroughly discussed in the previous sections of this paper, only ACNs' shortcomings are described below.

First and foremost, local antibiotic carriers have never been shown to be superior to intravenous administration of antibiotics in terms of cure rate. At the same time, most of them require some sort of repeat surgery, unless they biodegrade, which is not the case in ACNs [44].

There has been one report linking high failure rate to the use of ACNs in infected nonunion with bone defect exceeding 6 cm [34]. The authors suggested that when faced with a large bone defect, other alternative forms of treatment should be used. Unfortunately, they did not provide a multivariate analysis on risk factors that might influence their results.

One of the concerns with all the PMMA carriers for antibiotic is the emergence of resistance. There have been reports on induction of coagulase-negative staphylococci after applying gentamicin-loaded bone cement [69]. However, there are, to our knowledge, no reports on resistance after ACNs.

From the technical point of view, there is a possibility of debonding of the cement from the nail upon its introduction or removal from the medullary canal. However, this situation is usually quickly resolved with reaming or using long-reaching surgical tools and, in our experience, as with every surgical complication, the rate of debonding decreases with the familiarity with the technique and increasing expertise in nail fabrication [23, 46].

In the past, bone cement liquid monomer, methyl methacrylate (MMA), was believed to be carcinogenic. However, the lack of consistency in the results of various studies and the absence of dose response lead to the conclusion that MMA in not carcinogenic to humans under normal conditions of use [104]. Moreover, the evidence shows that even repeated mixing of PMMA bone cement does not pose an additional risk to operative theatres' personnel [105]. In a study comparing ionically dissolved or precipitated metals with MMA toxicity, MMA monomer toxicity was found to be low compared to metal toxicity [47]. The study of Elmaraghy et al. found that the presence of MMA monomer in femoral venous blood has no effect on the formation of fat emboli or their pulmonary haemodynamic outcome during cemented hip arthroplasty [106]. To our knowledge, there are no studies on MMA toxicity after the use of ACNs.

### 3.11. Alternatives

The novel concept of local antibiotic delivery by gentamicin-impregnated acrylic bone cement was introduced by Buchholz et al. in the early 1970s for the treatment of infected hip arthroplasty [65]. Antibiotic bead chains were introduced by Klemm in 1976 [24]. Later on, his team reported on the use of an antibiotic-impregnated cement “stick” [25, 107].

In a study of two cases, Tandon and Thomas were able to provide a proof of concept of using hollow, slotted nail with gentamicin cement beads. Based on the observation that commercially available beads are 7 mm in diameter, they argued that two to three strings of beads could be inserted into most intramedullary nails and effectively treat infection [108]. Unfortunately, their technique did not allow for the use of interlocking screws, as they would make extracting the beads without the nail impossible, and was never published on a larger series.

### 3.12. Outlook

It is perceived that with future developments local antibiotic delivery systems will likely supplant the traditional use of systemic antibiotics for the treatment of musculoskeletal infections [7, 109].

Another field of development is the MRI-compatible ACN. There has been only a single, short follow-up case report by Mauffrey et al. The authors claim that serum inflammatory markers might not always be reliable and that MRI monitoring of the infection might provide better insight into whether or not the infection has healed and one could move to the definitive fixation by a metallic nail [36]. On the other hand, the use of carbon fibre nail was estimated to incur an additional cost of $2,600, which is almost 30-fold higher than for most of the nails described in the literature [23].

Since 2005, there are also prefabricated interlocking antibiotic-coated titanium nails, which use polylactic acid (PLA) coating loaded with gentamicin and offer both sustained release kinetics and biodegradability. Promising 6-month results have been reported in primary fracture setting [110].

It is important to realize that as long as none of the ACNs is FDA-approved, which is the current state as of 2015, their use is off-label and prospective clinical trials cannot be undertaken [44].

There is only one short paediatric case series, in which ACNs have been used. It was found to be safe and effective in the treatment of chronic osteomyelitis, with special attention to protecting the epiphyseal growth plates of long bones. The authors believed also that using the nail instead of other forms of local antibiotic carrier enabled for prevention of further tissue loss [33]. We believe this patient group could benefit from the use of ACNs, but further clinical trials are necessary before introducing the ACNs into regular paediatric trauma/orthopaedic practice.

There are certain substances other than antibiotics, such as dextran, glycine, or xylitol, which could be used to impregnate PMMA for the enhancement of the antibiotic elution. However, the ideal filler material and amount of filler are yet to be established [8]. We are not aware of any studies beyond the laboratory phase on the PMMA fillers [111].

Another technical development is the introduction of the Reamer-Irrigator-Aspirator (RIA) system (Synthes, Inc., West Chester, Philadelphia) which, in animal models and cadaver studies, offers an advance on existing reaming devices, as it is less traumatic [32, 42]. It is used in the debridement and irrigation of the intramedullary canal of the femur and tibia [112]. Its use is very promising and could facilitate proper debridement of the medullary canal, which is the basic condition of intramedullary infection healing.

Ultrasound was also found to increase the transport of gentamicin across and within the biofilm of* P. aeruginosa* and* E. coli*. The other effect of ultrasound waves is presumably to increase the transport of oxygen and other small molecules, which may increase the metabolic state and render cells more susceptible to antibiotics [113, 114]. The ultrasound may have also a positive effect on antibiotic elution characteristics [8]. Further laboratory, feasibility, and clinical studies are needed to explain the potential for enhanced antibiotic release from PMMA by ultrasound and translate those basic science findings into clinical practice.

## 4. Conclusions

Since the introduction of antibiotic loaded acrylic bone cement by Buchholz and Engelbrecht, it remains a golden standard in local antibiotic delivery. This is also true in case of long bone medullary infections, where antibiotic cement nails remain an important treatment option. The major advantage of ACNs is the local release of high antibiotic concentrations, which vastly exceed those after systemic administration with no or low systemic toxicity. At the same time, they are able to provide limited stability to the fracture/nonunion site, which promotes infection healing. They are used as a clinician directed application; therefore, there is no issue with availability and antibiotic specificity, as it can be based on preoperative culture. Antibiotic elution rates, mechanisms, and ways to improve total amount of drug delivered are still debated and researched. Despite promising short- and midterm results from clinical studies, further basic science and translational studies are desirable before routine use of ACNs in clinical practice.

## Figures and Tables

**Table 1:** Literature review of antibiotic cement nail cases.

First author	Year	Number of cases	Anatomical site	Mean age (range)	Follow-up (range)	Nail formulation per 40 g PMMA	Results
Case series							
Kanakaris [28]	2014	24	14 femurs 10 tibias	47 (17–75) years	19 (8–36) months	0.5 g gentamicin 2 g vancomycin, antifungals	4% recurrence 5% failure in nail removal
Asloum [29]	2014	28	3 femurs 25 tibias	43 (19–70) years	48 (4–96) months	Unknown	7% recurrence 7% complications
Dhanasekhar [30]	2013	18	Femurs and tibias	Unknown	Unknown	2 g vancomycin 0.5 gentamicin	0% recurrence
Wasko [23]	2013	10	10 tibias	42 (20–59) years	72 (60–84) months	2.5 gentamicin	0% recurrence 0% complications
Selhi [31]	2011	16	8 femurs 7 tibias 1 humerus	38 (18–54) years	Unknown	4 g vancomycin 0.5 gentamicin	31% recurrence 0% complications
Kanakaris [32]	2011	8	8 femurs	40 (22–76) years	12 (12–20) months	0.5 g gentamicin 2 g vancomycin	0% recurrence 0% complications
Bar-On [33]	2010	4	2 femurs 2 tibias	9 (5–14) years	41 (36–46) months	Unknown	0% recurrence 50% wound healing disturbances
Bhadra [13]	2009	30	Lower limb	47 (20–79) years	26 (4–40) months	1.2 mg tobramycin 1 g vancomycin	Unknown
Shyam [34]	2009	25	23 femurs 2 tibias	33 (21–58) years	29 (18–40) months	2 g vancomycin 2 g gentamicin	20% recurrence 0% complications
Sancineto [27]	2008	18	4 femurs 14 tibias	37 (18–52) years	12 (10–54) months	4 g vancomycin Various others	6% recurrence 6% intolerance to nail
Qiang [35]	2007	19	5 femurs 14 tibias	38 (22–78) years	16 (6–28) months	2 g vancomycin	0% recurrence 5% complications
Paley [21]	2002	9	6 femurs 2 tibias 1 humerus	30 (8–70) years	41 (32–48) months	2.4 tobramycin 2 g vancomycin	0% recurrence 10% rod fracture

Case reports							
Mauffrey [36]	2014	1	1 femur	58 years	2 months	0.5 gentamicin 2.4 g tobramycin 2 g vancomycin	Unknown
Riel [37]	2010	1	1 tibia	±65 years	Unknown	0.5 g gentamicin	Unknown
Madanagopal [12]	2004	1	1 tibia	Unknown	Unknown	1.2 g tobramycin	0% recurrence
Ohtsuka [38]	2002	1	1 tibia	28 years	18 months	1.2 gentamicin	0% recurrence

**Table 2:** The benefits and drawbacks of antibiotic cement nails.

Benefits	Drawbacks
High concentration of local antibiotic elution: up to 200 times greater than with systemic drug administration, independent of vascular supply [21] Stability to the fracture/nonunion site, allowing for early weight bearing [23] Local antibiotic delivery independent of patient compliance [23] Systemic toxicity of antibiotics very rarely observed [8] Versatility of modifying antibiotic as per the culture report [27] Control of infection and stability is achieved with a single procedure [44] Alternative for patients refusing or not being right candidates for external fixation [13]	Local antibiotic carriers have never been shown to be superior to intravenous administration of antibiotics in terms of cure rate [21] Require repeat surgery [23] Possible emergence of resistance [13] Possible MMA toxicity [102, 103]
